# A Value and Ambiguity-Based Ranking Method of Trapezoidal Intuitionistic Fuzzy Numbers and Application to Decision Making

**DOI:** 10.1155/2014/560582

**Published:** 2014-07-22

**Authors:** Xiang-tian Zeng, Deng-feng Li, Gao-feng Yu

**Affiliations:** ^1^Institute of Tourism, Sanming University, Sanming, Fujian 365004, China; ^2^School of Economics and Management, Fuzhou University, Fuzhou, Fujian 350108, China; ^3^Institute of Information Engineering, Sanming University, Sanming, Fujian 365004, China

## Abstract

The aim of this paper is to develop a method for ranking trapezoidal intuitionistic fuzzy numbers (TrIFNs) in the process of decision making in the intuitionistic fuzzy environment. Firstly, the concept of TrIFNs is introduced. Arithmetic operations and cut sets over TrIFNs are investigated. Then, the values and ambiguities of the membership degree and the nonmembership degree for TrIFNs are defined as well as the value-index and ambiguity-index. Finally, a value and ambiguity-based ranking method is developed and applied to solve multiattribute decision making problems in which the ratings of alternatives on attributes are expressed using TrIFNs. A numerical example is examined to demonstrate the implementation process and applicability of the method proposed in this paper. Furthermore, comparison analysis of the proposed method is conducted to show its advantages over other similar methods.

## 1. Introduction

Multiattribute decision making (MADM) is an important research field of decision science, operational research, and management science. MADM is the process of identifying the problem, constructing the preferences, evaluating the alternatives, and determining the best alternatives. The classical decision making methods assume that accurate data is available to determine the best alternatives among the available options. However, in practice, due to the inherent uncertainty and impression of the available data, it is often impossible to obtain accurate information. Therefore, decision making under fuzzy environment problem is an interesting research topic having received more and more attention from researchers during the last several years.

The fuzzy set [1] was extended to develop the intuitionistic fuzzy (IF) set [2, 3] by adding an additional nonmembership degree, which may express more abundant and flexible information as compared with the fuzzy set [4–6]. Fuzzy numbers are a special case of fuzzy sets and are of importance for fuzzy multiattribute decision making problems [7–12]. As a generalization of fuzzy numbers, an IFN seems to suitably describe an ill-known quantity [13].

For decision making using the IF sets, it is required to rank the IFNs. So far, several methods have been developed for ranking the IFNs. Mitchell [14] interpreted IFNs as an ensemble of ordinary fuzzy numbers and defined a characteristic vagueness factor and a ranking method for IFNs. Nan et al. [15] defined the concept of average indexes for ranking triangular IFNs. Nehi [16] generalized the concept of characteristic value introduced for the membership and the nonmembership functions and proposed a ranking method based on this concept. Mitchell [14] interpreted an IFN as an ensemble of fuzzy numbers and introduced a ranking method. Nayagam et al. [17]. described IFNs of a special type and introduced a method of IFNs scoring that generalized Chen and Hwang's scoring for ranking IFNs. Grzegrorzewski [18] defined IFNs of a particular type and proposed a ranking method by using the expected interval of an IFN. By adding a degree of nonmembership, Shu et al. [19] defined a triangular IFN (TIFN) in a similar way to the fuzzy number introduced by Li [13] and developed an algorithm for IF fault tree analysis. Wang and Zhang [20] defined the TIFNs and gave a ranking method which transformed the ranking of TIFNs into the ranking of interval numbers.

In this paper, TrIFNs are introduced as a special type of IFNs, which have appealing interpretations and can be easily specified and implemented by the decision maker. The concept of the TrIFNs and ranking method as well as applications are discussed in depth.

This paper is organized as follows. In Section 2, the concepts of TrIFNs and cut sets as well as arithmetical operations are introduced.  Section 3 defines the concepts of the value and ambiguity of the membership and nonmembership functions as well as the value index and ambiguity index. Hereby a ranking method is developed for ranking TrIFNs.  Section 4 formulates MADM problems with TrIFNs, which are solved by the extended simple weighted average method using the ranking method proposed in this paper. A numerical example and a comparison analysis are given in Section 5. This paper concludes in Section 6.

## 2. Basic Definitions

### 2.1. The Definition and Operations of TrIFNs

**Definition 1 .**

**Figure 1:**
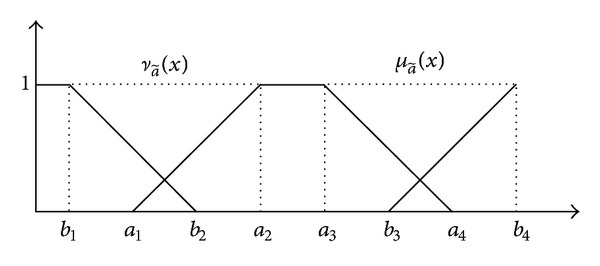
TrIFN.

A TrIFN $\tilde{a}=(a_{1},a_{2},a_{3},a_{4};b_{1},b_{2},b_{3},b_{4})$ is a special IF set on the real number set *R*, whose membership function and nonmembership function are defined as follows:

(1) $$\begin{array}{c} \mu_{\tilde{a}}\left(x\right)=\{\begin{array}{cc} 0 & \left(x⩽a_{1}\right)\\ \frac{x-a_{1}}{a_{2}-a_{1}} & \left(a_{1}⩽x⩽a_{2}\right)\\ 1 & \left(a_{2}⩽x⩽a_{3}\right)\\ \frac{a_{4}-x}{a_{4}-a_{3}} & \left(a_{3}⩽x⩽a_{4}\right)\\ 0 & \left(a_{4}⩽x\right), \end{array} \end{array}$$

(2) $$\begin{array}{c} \nu_{\tilde{a}}\left(x\right)=\{\begin{array}{cc} 1 & \left(x⩽b_{1}\right)\\ \frac{b_{2}-x}{b_{2}-b_{1}} & \left(b_{1}⩽x⩽b_{2}\right)\\ 0 & \left(b_{2}⩽x⩽b_{3}\right)\\ \frac{x-b_{3}}{b_{4}-b_{3}} & \left(b_{3}⩽x⩽b_{4}\right)\\ 1 & \left(b_{4}⩽x\right), \end{array} \end{array}$$

respectively, where *b*_1_ ⩽ *a*_1_, *b*_2_ ⩽ *a*_2_, *a*_3_ ⩽ *b*_3_, and *a*_4_ ⩽ *b*_4_. The membership and nonmembership functions of TrINF $\tilde{a}$ are illustrated in Figure 1.

If *a*_2_ = *a*_3_ and *b*_2_ = *b*_3_, an TrIFN $\tilde{a}$ degenerates to TIFN. Hence, the TIFNs are considered as special cases of the TrINFs.

In a similar way to arithmetic operations of IFNs, the arithmetic operations of TrINFs can be defined as follows.

**Definition 2 .**

Let ${\tilde{a}}_{1}=(a_{11},a_{12},a_{13},a_{14};b_{11},b_{12},b_{13},b_{14})$ and ${\tilde{a}}_{2}=(a_{21},a_{22},a_{23},a_{24};b_{21},b_{22},b_{23},b_{24})$ be two TrIFNs, where *a*_*ij*_ > 0 and *b*_*ij*_ > 0 (*i* = 1, 2; *j* = 1, 2, 3, 4), and let *λ* be any positive real number. The arithmetic operations over TrIFNs are defined as follows:

1. ${\tilde{a}}_{1}+{\tilde{a}}_{2}=(a_{11}+a_{21},a_{12}+a_{22},a_{13}+a_{23}$, *a*_14_ + *a*_24_; *b*_11_ + *b*_21_, *b*_12_ + *b*_22_, *b*_13_ + *b*_23_, *b*_14_ + *b*_24_);
2. ${\tilde{a}}_{1}-{\tilde{a}}_{2}=(a_{11}-a_{21},a_{12}-a_{22},a_{13}-a_{23}$, *a*_14_ − *a*_24_; *b*_11_ − *b*_21_, *b*_12_ − *b*_22_, *b*_13_ − *b*_23_, *b*_14_ − *b*_24_);
3. ${\tilde{a}}_{1}{\tilde{a}}_{2}=(a_{11}a_{21},a_{12}a_{22},a_{13}a_{23},a_{14}a_{24};b_{11}b_{21},b_{12}b_{22}$, *b*_13_ *b*_23_, *b*_14_ *b*_24_);
4. ${\tilde{a}}_{1}/{\tilde{a}}_{2}=(a_{11}/a_{24},a_{12}/a_{23},a_{13}/a_{22},a_{14}/a_{21};b_{11}/b_{24}$, *b*_12_/*b*_23_, *b*_13_/*b*_22_, *b*_14_/*b*_21_);
5. $\lambda {\tilde{a}}_{1}=(\lambda a_{11},\lambda a_{12},\lambda a_{13},\lambda a_{14};\lambda b_{11},\lambda b_{12},\lambda b_{13},\lambda b_{14})$;
6. ${\tilde{a}}_{1}^{\lambda }=(a_{11}^{\lambda },a_{12}^{\lambda },a_{13}^{\lambda },a_{14}^{\lambda };b_{11}^{\lambda },b_{12}^{\lambda },b_{13}^{\lambda },b_{14}^{\lambda })$.

From Definition 2, the following properties are proven:

1. ${\tilde{a}}_{1}+{\tilde{a}}_{2}={\tilde{a}}_{2}+{\tilde{a}}_{1}$, ${\tilde{a}}_{1}{\tilde{a}}_{2}={\tilde{a}}_{2}{\tilde{a}}_{1}$;
2. $\lambda ({\tilde{a}}_{1}+{\tilde{a}}_{2})=\lambda {\tilde{a}}_{1}+\lambda {\tilde{a}}_{2}$, $\;\lambda_{1}{\tilde{a}}_{1}+\lambda_{2}{\tilde{a}}_{1}=(\lambda_{1}+\lambda_{2}){\tilde{a}}_{1}$, $\;{\tilde{a}}_{1}^{\lambda_{1}+\lambda_{2}}={\tilde{a}}_{1}^{\lambda_{1}}{\tilde{a}}^{\lambda_{2}}$, where *λ*⩾0, *λ*_1_0 and *λ*_2_0;
3. ${\left({\tilde{a}}_{1}^{\lambda }\right)}^{k}={\tilde{a}}_{1}^{\lambda k}$, where ${\tilde{a}}_{1}>0$, *λ*⩾0 and *k*⩾0.

### 2.2. Cut Sets of TrIFNs

According to the cut sets of the IF set defined in [3], the cut sets of an TrIFN can be defined as follows.

**Definition 3 .**

An (*α*, *β*)-cut set of $\tilde{a}$ is a crisp subset *R*, which is defined as follows:

(3) $$\begin{array}{c} {\tilde{a}}_{\alpha ,\beta }=\left\{x\mu_{\tilde{a}}\left(x\right)⩾\alpha ,\;\;\nu_{\tilde{a}}\left(x\right)⩽\beta \right\}, \end{array}$$

where 0 ⩽ *α* ⩽ 1, 0 ⩽ *β* ⩽ 1, and 0 ⩽ *α* + *β* ⩽ 1.

**Definition 4 .**

A *α*-cut set of $\tilde{a}$ is a crisp subset *R*, which is defined as follows:

(4) $$\begin{array}{c} {\tilde{a}}_{\alpha }=\left\{x\;|\;\mu_{\tilde{a}}\left(x\right)⩾\alpha \right\}. \end{array}$$

Using (3) and Definition 4, it follows that ${\tilde{a}}_{\alpha }$ is closed interval, denoted by ${\tilde{a}}_{\alpha }=[L^{\alpha }(\tilde{a}),R^{\alpha }(\tilde{a})]$, which can be calculated as follows:

(5) $$\begin{array}{c} \left[L_{\tilde{a}}\left(\alpha \right),R_{\tilde{a}}\left(\alpha \right)\right]=\left[a_{1}+\alpha \left(a_{2}-a_{1}\right),a_{4}-\alpha \left(a_{4}-a_{3}\right)\right]. \end{array}$$

**Definition 5 .**

A *β*-cut set of $\tilde{a}$ is a crisp subset *R*, which is defined as follows:

(6) $$\begin{array}{c} {\tilde{a}}_{\beta }=\left\{x\;|\;\nu_{\tilde{a}}\left(x\right)⩽\beta \right\}. \end{array}$$

Using (4) and Definition 5, it follows that ${\tilde{a}}_{\beta }$ is closed interval, denoted by ${\tilde{a}}_{\beta }=[L^{\beta }(\tilde{a}),R^{\beta }(\tilde{a})]$, which can be calculated as follows:

(7) $$\begin{array}{c} \left[L_{\tilde{a}}\left(\beta \right),R_{\tilde{a}}\left(\beta \right)\right]=\left[b_{2}-\beta \left(b_{2}-b_{1}\right),b_{3}+\beta \left(b_{4}-b_{3}\right)\right]. \end{array}$$

## 3. Characteristic of TrIFNs and the Value and Ambiguity-Based Ranking Method

### 3.1. Value and Ambiguity of TrIFNs

In this section, the value and ambiguity of TrIFNs are defined as follows.

**Definition 6 .**

Let ${\tilde{a}}_{\alpha }$ and ${\tilde{a}}_{\beta }$ be any *α*-cut and *β*-cut set of an TrFN $\tilde{a}$, respectively. The value of the membership function $\mu_{\tilde{a}}(x)$ and nonmembership function $\nu_{\tilde{a}}(x)$ for the TrIFN $\tilde{a}$ is defined as follows:

(8) $$\begin{array}{c} V_{\mu }=\overset{1}{\underset{0}{\int }}\left(L_{\tilde{a}}\left(\alpha \right)+R_{\tilde{a}}\left(\alpha \right)\right)f\left(\alpha \right)d\alpha , \end{array}$$

(9) $$\begin{array}{c} V_{\nu }=\overset{1}{\underset{0}{\int }}\left(L_{\tilde{a}}\left(\beta \right)+R_{\tilde{a}}\left(\beta \right)\right)g\left(\beta \right)d\beta , \end{array}$$

respectively.

The function *f*(*α*) = *α* gives different weights to elements in different *α*-cut sets. In fact, *α* diminishes the contribution of the lower *α*-cut sets, which is reasonable since these cut sets arising from values of $\mu_{\tilde{a}}(x)$ have a considerable amount of uncertainty. Obviously, $V_{\mu }(\tilde{a})$ synthetically reflects the information on every membership degree and may be regarded as a central value that represents from the membership function point of view. Similarly, the function *g*(*β*) = 1 − *β* has the effect of weighting on the different *β*-cut sets. *g*(*β*) diminishes the contribution of the higher *β*-cut sets, which is reasonable since these cut sets arising from values of $\nu_{\tilde{a}}(x)$ have a considerable amount of uncertainty. $V_{\nu }(\tilde{a})$ synthetically reflects the information on every nonmembership degree and may be regarded as a central value that represents from the nonmembership function point of view.

According to (8), the value of the membership function of a TrIFN $\tilde{a}$ is calculated as follows:

(10) Vμ=∫01(La~(α)+Ra~(α))f(α)dα=∫01[a1+α(a2−a1)+a4−α(a4−a3)]α dα=16(a1+2a2+2a3+a4);

that is,

(11) Vμ=16(a1+2a2+2a3+a4).

In a similar way, according to (9), the value of the nonmembership function of a TrIFN $\tilde{a}$ is calculated as follows:

(12) Vν=∫01(La~(β)+Ra~(β))g(β)dβ=∫01[b2−β(b2−b1)+b3+β(b4−b3)](1−β)dβ=16(b1+2b2+2b3+b4);

that is,

(13) Vν=16(b1+2b2+2b3+b4).

**Definition 7 .**

Let ${\tilde{a}}_{\alpha }$ and ${\tilde{a}}_{\beta }$ be any *α*-cut and *β*-cut set of an TrIIFN $\tilde{a}$, respectively. The ambiguity of the membership function $\mu_{\tilde{a}}(x)$ and nonmembership function $\nu_{\tilde{a}}(x)$ for the TrIIFN $\tilde{a}$ is defined as follows:

(14) $$\begin{array}{c} A_{\mu }=\overset{1}{\underset{0}{\int }}\left(R_{\tilde{a}}\left(\alpha \right)-L_{\tilde{a}}\left(\alpha \right)\right)f\left(\alpha \right)d\alpha , \end{array}$$

(15) $$\begin{array}{c} A_{\nu }=\overset{1}{\underset{0}{\int }}\left(R_{\tilde{a}}\left(\beta \right)-L_{\tilde{a}}\left(\beta \right)\right)g\left(\beta \right)d\beta . \end{array}$$

It is easy to see that $R_{\tilde{a}}(\alpha )-L_{\tilde{a}}(\alpha )$ and $R_{\tilde{a}}(\beta )-L_{\tilde{a}}(\beta )$ are just about the lengths of the intervals ${\tilde{a}}^{\alpha }$ and ${\tilde{a}}_{\beta }$, respectively. Thus, $A_{\mu }(\tilde{a})$ and $A_{\nu }(\tilde{a})$ can be regarded as the global spreads of the membership function $\mu_{\tilde{a}}$ and the nonmembership function $\nu_{\tilde{a}}(x)$. Obviously, $A_{\mu }(\tilde{a})$ and $A_{\nu }(\tilde{a})$ basically measure how much there is vagueness in the $\tilde{a}(x)$.

According to (14), the ambiguity of the membership function of a TrIFN $\tilde{a}$ is calculated as follows:

(16) Aμ=∫01(Ra~(α)−La~(α))f(α)dα=∫01[a4−α(a4−a3)−a1−α(a2−a1)]α dα=16(−a1−2a2+2a3+a4);

that is,

(17) Aμ=16(−a1−2a2+2a3+a4).

Likewise, according to (15), the ambiguity of the nonmembership function of a TrIFN $\tilde{a}$ is calculated as follows:

(18) Aν=∫01(Ra~(β)−La~(β))g(β)dβ=∫01[b3+β(b4−b3)−b2+β(b2−b1)](1−β)dβ=16(−b1−2b2+2b3+b4);

that is,

(19) Aν=16(−b1−2b2+2b3+b4).

### 3.2. The Value and Ambiguity-Based Ranking Method

Based on the above value and ambiguity of a TrIFN, a new ranking method of TrIFNs is proposed in this subsection. A value-index and an ambiguity-index for $\tilde{a}$ are firstly defined as follows.

**Definition 8 .**

Let $\tilde{a}=(a_{1},a_{2},a_{3},a_{4};b_{1},b_{2},b_{3},b_{4})$ be a TrIFN. A value-index and ambiguity-index for $\tilde{a}$ are defined as follows:

(20) $$\begin{array}{c} V_{\lambda }\left(\tilde{a}\right)=\lambda V_{\mu }\left(\tilde{a}\right)+\left(1-\lambda \right)V_{\nu }\left(\tilde{a}\right), \end{array}$$

(21) $$\begin{array}{c} A_{\lambda }\left(\tilde{a}\right)=\lambda A_{\mu }\left(\tilde{a}\right)+\left(1-\lambda \right)A_{\nu }\left(\tilde{a}\right), \end{array}$$

respectively, where *λ* ∈ [0,1] is a weight which represents the decision maker's preference information.

*λ* ∈ (0.5,1] shows that the decision maker prefers uncertainty or negative feeling; *λ* ∈ [0,0.5) shows that the decision maker prefers certainty or positive feeling; *λ* = 0.5 shows that the decision maker is indifferent between positive feeling and negative feeling. Therefore, the value index and the ambiguity index may reflect the decision maker's subjectivity attitude to the TrIFNs.

It is easily seen that $V_{\lambda }(\tilde{a})$ and $A_{\lambda }(\tilde{a})$ have some useful properties, which are summarized in Theorems 9 and 10.

**Theorem 9 .**

Let ${\tilde{a}}_{1}=(a_{11},a_{12},a_{13},a_{14};b_{11},b_{12},b_{13},b_{14})$ and ${\tilde{a}}_{2}=(a_{21},a_{22},a_{23},a_{24};b_{21},b_{22},b_{23},b_{24})$ be two TrIFNs. Then for any *λ* ∈ (0,1] and *γ* ∈ *R* ^+^, the following equation is valid:

(22) $$\begin{array}{c} V_{\lambda }\left({\tilde{a}}_{1}+{\tilde{a}}_{2}\right)=V_{\lambda }\left({\tilde{a}}_{1}\right)+V_{\lambda }\left({\tilde{a}}_{2}\right),\\ V_{\lambda }\left(\gamma {\tilde{a}}_{1}\right)=\gamma V_{\lambda }\left({\tilde{a}}_{1}\right). \end{array}$$

**Proof**

According to Definition 2, we have

(23) a~1+a~2=(a11+a21,a12+a22,a13+a23,a14+a24; b11+b21,b12+b22,b13+b23,b14+b24).

Using (11), (13), and (20), we obtain

(24) Vλ(a~1+a~2)=λVμ(a~1+a~2)+(1−λ)Vν(a~1+a~2)=λ16[(a11+a21)+2(a12+a22)   +2(a13+a23)+(a14+a24)] +(1−λ)16[(b11+b21)+2(b12+b22)        +2(b13+b23)+(b14+b24)]=λ16(a11+2a12+2a13+a14) +(1−λ)16(b11+2b12+2b13+b14) ×λ16(a21+2a22+2a23+a24) +(1−λ)16(b21+2b22+2b23+b24)=Vλ(a~1)+Vλ(a~2);

that is, $V_{\lambda }({\tilde{a}}_{1}+{\tilde{a}}_{2})=V_{\lambda }({\tilde{a}}_{1})+V_{\lambda }({\tilde{a}}_{2})$.

Furthermore, from

(25) $$\begin{array}{c} \lambda {\tilde{a}}_{1}=\left(\lambda a_{11},\lambda a_{12},\lambda a_{13},\lambda a_{14};\lambda b_{11},\lambda b_{12},\lambda b_{13},\lambda b_{14}\right). \end{array}$$

Using (11), (13), and (20), we get

(26) Vλ(γa~1)=λVμ(γa~1)+(1−λ)Vν(γa~1)=λ16(γa11+2γa12+2γa13+γa14) +16(1−λ)(γb11+2γb12+2γb13+γb14)=γ[λ16(a11+2a12+2a13+a14)  +16(1−λ)(b11+2b12+2b13+b14)]=γVλ(a~1).

That is, $V_{\lambda }(\gamma {\tilde{a}}_{1})=\gamma V_{\lambda }({\tilde{a}}_{1})$.

**Theorem 10 .**

Let ${\tilde{a}}_{1}=(a_{11},a_{12},a_{13},a_{14};b_{11},b_{12},b_{13},b_{14})$ and ${\tilde{a}}_{2}=(a_{21},a_{22},a_{23},a_{24};b_{21}$, *b*_22_, *b*_23_, *b*_24_) be two TrIFNs. Then for any *λ* ∈ (0,1] and *γ* ∈ *R* ^+^, the following equation is valid:

(27) $$\begin{array}{c} A_{\lambda }\left({\tilde{a}}_{1}+{\tilde{a}}_{2}\right)=A_{\lambda }\left({\tilde{a}}_{1}\right)+A_{\lambda }\left({\tilde{a}}_{2}\right),A_{\lambda }\left(\gamma {\tilde{a}}_{1}\right)=\gamma A_{\lambda }\left({\tilde{a}}_{1}\right). \end{array}$$

**Proof**

According to Definition 2, we have

(28) a~1+a~2=(a11+a21,a12+a22,a13+a23,a14+a24;  b11+b21,b12+b22,b13+b23,b14+b24),λa~1=(λa11,λa12,λa13,λa14;λb11,λb12,λb13,λb14).

Using (14), (15), and (21), we obtain

(29) Aλ(a~1+a~2)=λAμ(a~1+a~2)+(1−λ)Aν(a~1+a~2)=λ16[−(a11+a21)−2(a12+a22)     +2(a13+a23)+(a14+a24)] +(1−λ)16[−(b11+b21)−2(b12+b22)        +2(b13+b23)+(b14+b24)]=λ16(−a11−2a12+2a13+a14) +(1−λ)16(−b11−2b12+2b13+b14) ×λ16(−a21−2a22+2a23+a24) +(1−λ)16(−b21−2b22+2b23+b24)=Aλ(a~1)+Aλ(a~2),Aλ(γa~1)=λAμ(γa~1)+(1−λ)Aν(γa~1)=λ16(−γa11−2γa12+2γa13+γa14) +16(1−λ)(−γb11−2γb12+2γb13+γb14)=γ[λ16(−a11−2a12+2a13+a14)  +16(1−λ)(−b11−2b12+2b13+b14)]=γAλ(a~1).

That is, $A_{\lambda }({\tilde{a}}_{1}+{\tilde{a}}_{2})=A_{\lambda }({\tilde{a}}_{1})+A_{\lambda }({\tilde{a}}_{2})$ and $A_{\lambda }(\gamma {\tilde{a}}_{1})=\gamma A_{\lambda }({\tilde{a}}_{1})$.

Let ${\tilde{a}}_{1}$ and ${\tilde{a}}_{2}$ be two TrIFNs. A lexicographic ranking procedure based on the value-index and ambiguity-index can be summarized as follows.

**Rule 1 .**

If $V_{\lambda }({\tilde{a}}_{1})<V_{\lambda }({\tilde{a}}_{2})$, then ${\tilde{a}}_{1}$ is smaller than ${\tilde{a}}_{2}$.

**Rule 2 .**

If $V_{\lambda }({\tilde{a}}_{1})>V_{\lambda }({\tilde{a}}_{2})$, then ${\tilde{a}}_{1}$ is greater than$\;\;{\tilde{a}}_{2}$.

**Rule 3 .**

If $V_{\lambda }({\tilde{a}}_{1})=V_{\lambda }({\tilde{a}}_{2})$ and $A_{\lambda }({\tilde{a}}_{1})>A_{\lambda }({\tilde{a}}_{2})$, then ${\tilde{a}}_{1}$ is smaller than$\;\;{\tilde{a}}_{2}$.

**Rule 4 .**

If $V_{\lambda }({\tilde{a}}_{1})=V_{\lambda }({\tilde{a}}_{2})$ and $A_{\lambda }({\tilde{a}}_{1})<A_{\lambda }({\tilde{a}}_{2})$, then ${\tilde{a}}_{1}$ is greater than$\;\;{\tilde{a}}_{2}$.

**Rule 5 .**

If $V_{\lambda }({\tilde{a}}_{1})=V_{\lambda }({\tilde{a}}_{2})$ and $A_{\lambda }({\tilde{a}}_{1})=A_{\lambda }({\tilde{a}}_{2})$, then ${\tilde{a}}_{1}$ is equal to ${\tilde{a}}_{2}$.

Wang and Kerre [21] proposed some axioms which are used to evaluate the rationality of a ranking method of fuzzy numbers. It is easy to verify that $V_{\lambda }(\tilde{a})$ satisfies the axioms *A*_1_–*A*_6_. Proofs that $V_{\lambda }(\tilde{a})$ satisfies the axioms *A*_1_–*A*_3_ and *A*_5_ are easily completed. In the following, we focus on verifying that $V_{\lambda }(\tilde{a})$ satisfies the axioms *A*_4_ and *A*_6_.

**Theorem 11 .**

Let ${\tilde{a}}_{1}$ and ${\tilde{a}}_{2}$ be two TrIFNs. If *a*_11_ > *a*_24_ and *b*_11_ > *b*_24_, then ${\tilde{a}}_{1}>{\tilde{a}}_{2}$.

**Proof**

It is derived from (11) that

(30) $$\begin{array}{c} V_{\mu }\left({\tilde{a}}_{1}\right)=\frac{1}{6}\left(a_{11}+2a_{12}+2a_{13}+a_{14}\right)>a_{11},\\ V_{\mu }\left({\tilde{a}}_{2}\right)=\frac{1}{6}\left(a_{21}+2a_{22}+2a_{23}+a_{24}\right)<a_{24}. \end{array}$$

Combining with *a*_11_ > *a*_24_, it directly follows that $V_{\mu }({\tilde{a}}_{1})>V_{\mu }({\tilde{a}}_{2})$.

Similarly, it follows that

(31) $$\begin{array}{c} V_{\nu }\left({\tilde{a}}_{1}\right)=\frac{1}{6}\left(b_{11}+2b_{12}+2b_{13}+b_{14}\right)>b_{11}, \end{array}$$

(32) $$\begin{array}{c} V_{\nu }\left({\tilde{a}}_{1}\right)=\frac{1}{6}\left(b_{21}+2b_{22}+2b_{23}+b_{24}\right)<b_{41}, \end{array}$$

respectively. Combining with *b*_11_ > *b*_24_, that $V_{\nu }({\tilde{a}}_{1})>V_{\nu }({\tilde{a}}_{2})$. Therefore,

(33) $$\begin{array}{c} \lambda V_{\mu }\left({\tilde{a}}_{1}\right)+\left(1-\lambda \right)V_{\nu }\left({\tilde{a}}_{1}\right)>\lambda V_{\mu }\left({\tilde{a}}_{2}\right)+\left(1-\lambda \right)V_{\nu }\left({\tilde{a}}_{2}\right), \end{array}$$

that is, $V_{\lambda }({\tilde{a}}_{1})>V_{\lambda }({\tilde{a}}_{2})$; hence, ${\tilde{a}}_{1}>{\tilde{a}}_{2}$.

**Theorem 12 .**

Let ${\tilde{a}}_{1}$ and ${\tilde{a}}_{2}$ be two TrIFNs. If  ${\tilde{a}}_{1}>{\tilde{a}}_{2}$, then ${\tilde{a}}_{1}+{\tilde{a}}_{3}>{\tilde{a}}_{2}+{\tilde{a}}_{3}$.

**Proof**

According to Theorem 10, we have

(34) $$\begin{array}{c} V_{\lambda }\left({\tilde{a}}_{1}+{\tilde{a}}_{3}\right)=V_{\lambda }\left({\tilde{a}}_{1}\right)+V_{\lambda }\left({\tilde{a}}_{3}\right). \end{array}$$

Similarly, it follows that

(35) $$\begin{array}{c} V_{\lambda }\left({\tilde{a}}_{2}+{\tilde{a}}_{3}\right)=V_{\lambda }\left({\tilde{a}}_{2}\right)+V_{\lambda }\left({\tilde{a}}_{3}\right) \end{array}$$

combining with ${\tilde{a}}_{1}>{\tilde{a}}_{2}$; then $V_{\lambda }({\tilde{a}}_{1})>V_{\lambda }({\tilde{a}}_{2})$. Then,

(36) $$\begin{array}{c} V_{\lambda }\left({\tilde{a}}_{1}+{\tilde{a}}_{3}\right)>V_{\lambda }\left({\tilde{a}}_{2}+{\tilde{a}}_{3}\right). \end{array}$$

Hence, ${\tilde{a}}_{1}+{\tilde{a}}_{3}>{\tilde{a}}_{2}+{\tilde{a}}_{3}$.

## 4. An Extended MADM Method Based on the Value and Ambiguity-Based Ranking Procedure

In this section, we will apply the above ranking method of TrIFNs to solve MADM problems in which the ratings of alternatives on attributes are expressed using TrIFNs. Sometimes such MADM problems are called as MADM problems with TrIFNs for short. Suppose that there exists an alternative set *A* = {*A*_1_, *A*_2_,…, *A*_*m*_}, which consists of *m* noninferior alternatives from which the most preferred alternative has to be selected. Each alternative is assessed on *n* attributes. Denote the set of all attributes by *X* = {*X*_1_, *X*_2_,…, *X*_*m*_}. Assume that ratings of alternatives on attributes are given using TrIFNs. Namely, the rating of any alternative *A*_*i*_ ∈ *A*  (*i* = 1, 2,…, *m*) on each attribute *X*_*j*_ ∈ *X*  (*j* = 1, 2,…, *n*) is an TrIFN ${\tilde{a}}_{ij}=(a_{ij1},a_{ij2},a_{ij3},a_{ij4};b_{ij1},b_{ij2},b_{ij3},b_{ij4})$. Thus, an MADM problem with TrIFNs can be expressed concisely in the matrix format as ${\left({\tilde{a}}_{ij}\right)}_{m\times n}$.

Due to the fact that different attributes may have different importance, assume that the relative weight of the attribute *x*_*j*_ is *w*_*j*_  (*j* = 1, 2,…, *n*), satisfying the normalization conditions: *w*_*j*_ ∈ [0,1] and ∑_*j*=1_^*n*^ *w*_*j*_ = 1. Let *w* = (*w*_1_, *w*_2_,…, *w*_*n*_)^*T*^ be the relative weight vector of all attributes. The extended additive weighted method for the MADM problem with TrIFNs can be summarized as follows.

**Step 1 .**

Identify the evaluation attitudes and alternatives.

**Step 2 .**

Pool the decision maker's opinion to get the ratings of alternatives on alternatives on attributes, that is, the TrIFN decision matrix $A={\left({\tilde{a}}_{ij}\right)}_{m\times n}$.

**Step 3 .**

Normalize the TrIFNs decision matrix. In order to eliminate the effect of different physical dimensions on the final decision making results, the normalized TrIFNs decision matrix can be calculated using the following formulae:

(37) r~ij=(aij1aj4+,aij2aj4+,aij3aj4+,aij4aj4+;bij1aj4+,bij2aj4+,bij3aj4+,bij4aj4+)  (i=1,2,…,m;  j∈B),

(38) r~ij=(bj1−aij4+,bj1−aij3+,bj1−aij2+bj1−aij1+;bj1−bij4+,bj1−bij3+bj1−bij2+,bj1−bij1+)(i=1,2,…,m;  j∈C),

respectively, where *B* and *C* are the subscript sets of benefit attributes and cost attributes, and *a*_*j*4_^+^ = max⁡⁡{*a*_*ij*4_∣*i* = 1,2,…, *m*}  (*j* ∈ *B*) and *b*_*j*1_^+^ = max⁡⁡{*b*_*ij*1_∣*i* = 1,2,…, *m*}  (*j* ∈ *C*).

**Step 4 .**

Calculate the weighted comprehensive values of alternatives. Using Definition 2, the weighted comprehensive values of alternatives *A*_*i*_ (*i* = 1, 2,…, *n*) are calculated as follows:

(39) $$\begin{array}{c} {\tilde{S}}_{i}=\overset{n}{\underset{j=1}{\sum }}w_{j}{\tilde{r}}_{ij}, \end{array}$$

respectively. Obviously, ${\tilde{S}}_{i}\;\;(i=1,2,\ldots ,m)$ are TrIFNs.

**Step 5 .**

Rank all alternatives. The ranking order of the alternatives *A*_*i*_ can be generated according to the nonincreasing order of the TrIFNs ${\tilde{S}}_{i}\;(i=1,2,\ldots ,m)$ by using the value and ambiguity- based ranking method proposed in Section 3.

## 5. An Application to an Investment Selection Problem and Comparison Analysis of the Results Obtained

### 5.1. An Investment Selection Problem and the Analysis Process

Let us suppose there is an investment company, which wants to invest a sum of money in best option. There is a panel with four possible to invert the money: *x*_1_ is a car company; *x*_2_ is a food company; *x*_3_ is a computer company; and *x*_4_ is a arms company. The investment company must take a decision according to the following three attitudes: *a*_1_ is the risk analysis; *a*_2_ is the growth analysis; and *a*_3_ is the environment impact analysis. The four possible alternatives *x*_*i*_  (*x* = 1, 2, 3, 4) are evaluated using the TrIFNs by decision maker under the above attributes, and the three attributes are benefit attributes; the weighted normalized TrIFNs decision matrix is obtained as shown in Table 1.

Using (39), the weighted comprehensive values of the candidates *x*_*i*_  (*i* = 1, 2, 3, 4) can be obtained as follows:

(40) $$\begin{array}{c} {\tilde{S}}_{1}=\left(0.22,0.32,0.42,0.52;0.13,0.32,0.42,0.62\right),\\ {\tilde{S}}_{2}=\left(0.44,0.54,0.64,0.74;0.35,0.54,0.64,0.82\right),\\ {\tilde{S}}_{3}=\left(0.37,0.47,0.57,0.67;0.28,0.47,0.57,0.78\right),\\ {\tilde{S}}_{4}=\left(0.46,0.56,0.66,0.76;0.40,0.56.0.66,0.82\right), \end{array}$$

respectively.

According to (11) and (13), the values of membership functions and nonmembership functions of ${\tilde{S}}_{1}$, ${\tilde{S}}_{2}$, ${\tilde{S}}_{3}$, and ${\tilde{S}}_{4}$ can be calculated as follows:

(41) $$\begin{array}{c} V_{\mu }\left({\tilde{S}}_{1}\right)=0.389,\;\;V_{\nu }\left({\tilde{S}}_{1}\right)=0.359;\\ V_{\mu }\left({\tilde{S}}_{2}\right)=0.588,\;\;V_{\nu }\left({\tilde{S}}_{2}\right)=0.566;\\ V_{\nu }\left({\tilde{S}}_{3}\right)=0.601,\;\;V_{\mu }\left({\tilde{S}}_{3}\right)=0.599;\\ V_{\mu }\left({\tilde{S}}_{4}\right)=0.600,\;\;V_{\nu }\left({\tilde{S}}_{4}\right)=0.582, \end{array}$$

respectively.

Using (20), the value-indices of ${\tilde{S}}_{1}$, ${\tilde{S}}_{2}$, ${\tilde{S}}_{3}$, and ${\tilde{S}}_{4}$ can be obtained as follows:

(42) Vλ(S~1)=0.359+0.031λ,  Vλ(S~2)=0.566+0.023λ,Vλ(S~3)=0.559+0.042λ,  Vλ(S~4)=0.582+0.017λ,

respectively.

It is easy to know that $V_{\lambda }({\tilde{S}}_{4})>V_{\lambda }({\tilde{S}}_{2})>V_{\lambda }({\tilde{S}}_{3})>V_{\lambda }({\tilde{S}}_{1})$ for any given weight *λ* ∈ [0,0.354) holds. Hence, the ranking order of the four companies is *x*_4_≻*x*_2_≻*x*_3_≻*x*_1_ if *λ* ∈ [0,0.354). In this case, the best selection is the company *x*_4_. However, if *λ* ∈ [0.354,0.947], then $V_{\lambda }({\tilde{S}}_{4})>V_{\lambda }({\tilde{S}}_{3})>V_{\lambda }({\tilde{S}}_{2})>V_{\lambda }({\tilde{S}}_{1})$, and the ranking order of the four companies is *x*_4_≻*x*_3_≻*x*_2_≻*x*_1_, and the best selection is the company *x*_4_. If *λ* ∈ [0.947,1] then $V_{\lambda }({\tilde{S}}_{3})>V_{\lambda }({\tilde{S}}_{4})>V_{\lambda }({\tilde{S}}_{2})>V_{\lambda }({\tilde{S}}_{1})$, and the ranking order of the four companies is *x*_3_≻*x*_4_≻*x*_2_≻*x*_1_, so the best selection is the company *x*_3_. Obviously, the ranking order of the four companies is related to the attitude parameter *λ* ∈ [0,1].

### 5.2. Comparison Analysis of the Results Obtained by the Ranking Method and Other Methods

To further illustrate the superiority of the decision method proposed in this paper, we apply some of the other methods to rank the TrIFNs ${\tilde{S}}_{1}$, ${\tilde{S}}_{2}$, ${\tilde{S}}_{3}$, and ${\tilde{S}}_{4}$. The ranking orders of ${\tilde{S}}_{1}$, ${\tilde{S}}_{2}$, ${\tilde{S}}_{3}$, and ${\tilde{S}}_{4}$ can be obtained as in Table 2.

From Table 1, if *λ* ∈ [0,0.354), then the ranking results obtained by the proposed method are the same as Nan's method, Nehi's method, and Wang's method. This shows that the proposed method is effective. However, the decision makers with different preference attitudes have different choices. Namely, a risk-taking decision maker may prefer *A*_3_, whereas a risk-averse decision maker may prefer *A*_4_. These factors cannot be reflected in Nan's method, Nehi's method, and Wang's method. Thus, the proposed method is more reasonable.

## 6. Conclusion

In this paper, we have studied two characteristics of a TrIFN, that is, the value and ambiguity, which are used to define the value index and ambiguity index of the TrIFN. Then, a ranking method is developed for the ordering of TrIFNs and applied to solve MADM problems with TrIFNs. Due to the fact that a TrIFN is a generalization of a trapezoidal fuzzy number, the other existing methods of ranking fuzzy numbers may be extended to TrIFNs. More effective ranking methods of TrIFNs will be investigated in the near future.

## Figures and Tables

**Table 1:** TrIFNs decision matrix.

	*a*_1_	*a*_2_	*a*_3_
*x*_1_	(0.26, 0.36, 0.46, 0.56;	(0.34, 0.44, 0.54, 0.64;	(0.12, 0.22, 0.32, 0.42;
	0.16, 0.36, 0.46, 0.66)	0.24, 0.44, 0.54, 0.74)	0.04, 0.22, 0.32, 0.50)
*x*_2_	(0.50, 0.60, 0.70, 0.80;	(0.30, 0.55, 0.70, 0.80;	(0.34, 0.44, 0.54, 0.54;
	0.42, 0.60, 0.70, 0.88)	0.22, 0.55, 0.70, 0.88)	0.34, 0.44, 0.54, 0.74)
*x*_3_	(0.55, 0.55, 0.68, 0.68;	(0.54, 0.64, 0.74, 0.84;	(0.36, 0.46, 0.56, 0.56;
	0.28, 0.55, 0.68, 0.78)	0.46, 0.54, 0.74, 0.92)	0.16, 0.46, 0.56, 0.66)
*x*_4_	(0.66, 0.76, 0.86, 0.96;	(0.55, 0.63, 0.78, 0.86;	(0.18, 0.28, 0.38, 0.48;
	0.64, 0.76, 0.86, 0.98)	0.55, 0.63, 0.78, 0.92)	0.08, 0.28, 0.38, 0.58)

**Table 2:** Ranking of TrIFNS using different methods.

Ranking methods	${\tilde{S}}_{1}$	${\tilde{S}}_{2}$	${\tilde{S}}_{3}$	${\tilde{S}}_{4}$	Ranking results
Nan et al. [15]	0.7425	1.1775	1.0425	1.22	*A*_4_≻*A*_2_≻*A*_3_≻*A*_1_
Nehi [16] (*k* = 0.5)	0.39	0.598	0.54	0.622	*A*_4_≻*A*_2_≻*A*_3_≻*A*_1_
Wang and Nie [22]	0.37125	0.58875	0.52125	0.61	*A*_4_≻*A*_2_≻*A*_3_≻*A*_1_
